# Retraction: Pleomorphic Adenoma of the Deep Lobe of the Parotid Gland Presenting With Dysphagia: A Case Report

**DOI:** 10.7759/cureus.r186

**Published:** 2025-07-16

**Authors:** Fatima M Farrh, Ahlam Alharbi

**Affiliations:** 1 Department of Otolaryngology, Head and Neck Surgery, University of Bahri, Khartoum, SDN; 2 Family Medicine, Primary Health Care Center, Riyadh, SAU

The Editors-in-Chief have retracted this article following a formal complaint and subsequent review. The radiological images were taken from a publicly available social media post without permission, and the article misrepresented patient details. The authors failed to respond to multiple requests for clarification. These issues compromise the article’s credibility and integrity, warranting its retraction.

